# PredictRx: AI based decision support tool for molecular screening for breast cancer drug recommendation

**DOI:** 10.3389/frai.2026.1881187

**Published:** 2026-07-28

**Authors:** Ritu Chauhan, Neha Pandey, Megat F. Zuhairi

**Affiliations:** 1 Artificial Intelligence and IoT Lab, Centre for Computational Biology and Bioinformatics, Amity University, Noida, India; 2 Malaysian Institute of Information Technology, Universiti Kuala Lumpur, Kuala Lumpur, Malaysia

**Keywords:** artificial intelligence, clustering, drug synergy, machine intelligence, web platform

## Abstract

**Introduction:**

Breast cancer remains one of the leading causes of cancer-related mortality rate worldwide, and the identification of effective drug combinations is an essential requirement in pharmaceutical research. The integration of Artificial Intelligence (AI) in processing large volumes of chemical and biological data combines molecular representation, predictive modeling and structured support within a single accessible tool, which accelerates early-stage candidate identification for breast cancer research while promoting reproducibility, transparency and user centered design.

**Aim:**

The current research focuses on developing and designing “PredictRx” which is an artificial intelligence based driven decision support tool which tends to benefit healthcare practioners to analyze the combination of drug which can be utilized for breast cancer patients.

**Methodology:**

PredictRx was developed using molecular descriptors, physicochemical properties, and drug interaction datasets collected from publicly available biomedical databases. The tool integrates in total six supervised and unsupervised learning techniques to examine the structural similarities between compounds and predict the potential drug interactions for breast cancer. Various machine learning techniques, including Random Forest, Support Vector Machine, Logistic Regression, K-Means Clustering, DBSCAN, and Agglomerative Clustering, to analyse structural similarities and predict potential drug interactions and synergy patterns. Model performance was evaluated using Classification matrix, Silhouette Score, Calinski-Harabasz Index, and Davies-Bouldin Index. The tool was deployed as a browser-accessible web application for real-time interaction and visualization.

**Result:**

The results suggests that Random Forest has the highest predictive performance accuracy of 1, and Agglomerative clustering delivered strongest scores (Silhouette Score: 0.6946; Davies-Bouldin Index: 0.2457). The current tool was deployed as a browser accessible web tool with possibility of real time interaction and result visualization. PredictRx is a distinctive easy to use, and interpretable screening tool focused on drug compatibility and synergy analysis. EDA further identified molecular weight, lipophilicity, and structural similarity as important contributors to drug compatibility prediction.

**Conclusion:**

PredictRx shows how AI-driven predictive modeling which can speed up molecular screening and early-stage breast cancer medication discovery. The technology facilitates the effective identification of appropriate drug combinations and offers a scalable foundation for upcoming AI-assisted pharmaceutical research by combining clustering, classification, molecular representation, and visualization into a single interpretable platform.

## 1 Introduction

Artificial Intelligence has emerged as a groundbreaking technology in biomedical research that allows processing the challenging biological information and helps make data driven decisions in a broad variety of areas of healthcare and life science. This technology allows the computational systems to give patterns in the big data sets, automate the analysis process as well as giving the forecasts which would not have been easily given in the traditional methods. AI in Biomedical research has become an integral component in diagnosis of diseases, genomic research, etc. With advanced algorithms and modeling techniques, the AI systems have higher processing capacity and factuality in terms of biological and chemical data, as they operate on large amounts of data (Chen et al., 2018; Vamathevan et al., 2019; Esteva et al., 2019; Topol, 2019). In addition to the constantly expanding supply of biological and chemical data, AI based computational technologies are rapidly emerging as an important tool in making the scientific discovery process faster and, moreover, improving the decision-making process within the pharmaceutical and biomedical research. The sphere of drug recommendation is one of the most important and urgent areas where these technologies have already become evident with the increasing popularity of AI-oriented computational assistance to support the process of identifying and optimizing therapeutic compounds (Vamathevan et al., 2019; Schneider et al., 2020; Bender and Cortés-Ciriano, 2021; Qureshi et al., 2023).

Developing new drugs is highly demanding and multi-step process involving target identification, lead identification, candidate molecule optimization, preclinical validation, and clinical testing. Traditionally, the pharmaceutical development cycle can take more than a decade and cost a significant amount of money before one drug candidate is finally introduced in the market. In addition, the percentage of candidate compounds that do not advance in early screening or clinical development due to poor therapeutic activity, unexpected toxicity or poor pharmacokinetic properties is very high. Nevertheless, with the advanced screening techniques, where the researcher is able to screen thousands of compounds, experimental screening remains expensive and logistically complex (Schneider et al., 2020; Bender and Cortés-Ciriano, 2021; Qureshi et al., 2023). In order to overcome these challenges, AI and ML tools are now commonly used to speed up early stage drug identification, cost reduction, computational screening, toxicity prediction, and optimization of therapies. By integrating predictive algorithms and cheminformatics, AI-based systems can offer insights into large chemical datasets, identify drug candidates with a high potential and save time and cost spent on early drug recommendation by far (Vamathevan et al., 2019; Sung et al., 2021; Harbeck et al., 2019; Brown et al., 2019; Pun et al., 2023).

Such computations have been helpful particularly in the issues of complicated diseases that require elaborate treatment protocols, where cancer has become one of the most prevalent issues of health among the general population in the globe. Even with the current discoveries in the medical field, the problem of breast cancer remains one of the biggest health challenges in the world. It is most prevalently diagnosed cancer among women and has been quoted one of the deadliest cancers globally with GLOBOCAN 2020 reporting over 2.3 million new diagnoses, which is the high-cost factor with regard to the healthcare system and social life (Lavecchia, 2015; Wigh et al., 2022). In addition to the clinical impact, breast cancer places major socioeconomic costs in terms of increased healthcare expenditures, productivity, and long-term survivorship care to patients and their households (Wigh et al., 2022). Subtypes of breast cancer are complicated and are characterized by biological differences and hence development of effective therapeutic options has been a substantial challenge which could require a great deal of experimentation and large-scale data analysis. In this regard, there has been a greater effort in employing the computational models and machine learning to find a way of quantifying the molecular data with predictions being made on the drug responses and other feasible therapeutic measures to treat breast cancer.

Artificial intelligence and informatics got to the stage when the complex nature of interaction of molecules and predicting the biological activity becomes much easier. Examples of machine learning algorithms applied in the biomedical field (drug recommendation, toxicity prediction, cancer research) and that are robust predictors include the well-known supervised algorithms like RF, SVM, and LR (Lavecchia, 2015; Hu et al., 2016; Bhatt et al., 2024). Furthermore, the computational models may be utilized in the modeling of important structural patterns of chemical compounds and also their potential biological effects via molecular representation schemes such as SMILES strings and also by utilizing chemical compound descriptors (Wigh et al., 2022; Hu et al., 2016; David et al., 2020; Winter et al., 2019). By such means, scientists can manage to filter a great number of compounds within the computer prior to it being testable and, thereby, this renders the research procedures more efficient and economical. Generative artificial intelligence and molecular design have significantly accelerated drug recommendation by enabling efficient exploration of chemical space and identification of novel compounds (Brown et al., 2019; Segler et al., 2018; Schwaller et al., 2021). Combining AlphaFold's detailed protein structures with the relational mapping from Graph Convolutional Networks (GCNs) opens up new possibilities for researchers. Now, they can see how a drug attaches to a specific protein “lock” and affects the body's intricate molecular web. This approach shifts drug recommendation away from focusing on individual molecules. Instead, it becomes an analysis of the entire system, predicting harmful side effects when multiple drugs interact by examining both structural changes and network-level impacts of such therapies (Jumper et al., 2021; Zitnik et al., 2018; Batool et al., 2019; Kearnes et al., 2016; Mayr et al., 2016; Altae-Tran et al., 2017; Nhlapho et al., 2024; Mak and Pichika, 2019).

**Figure 1:**
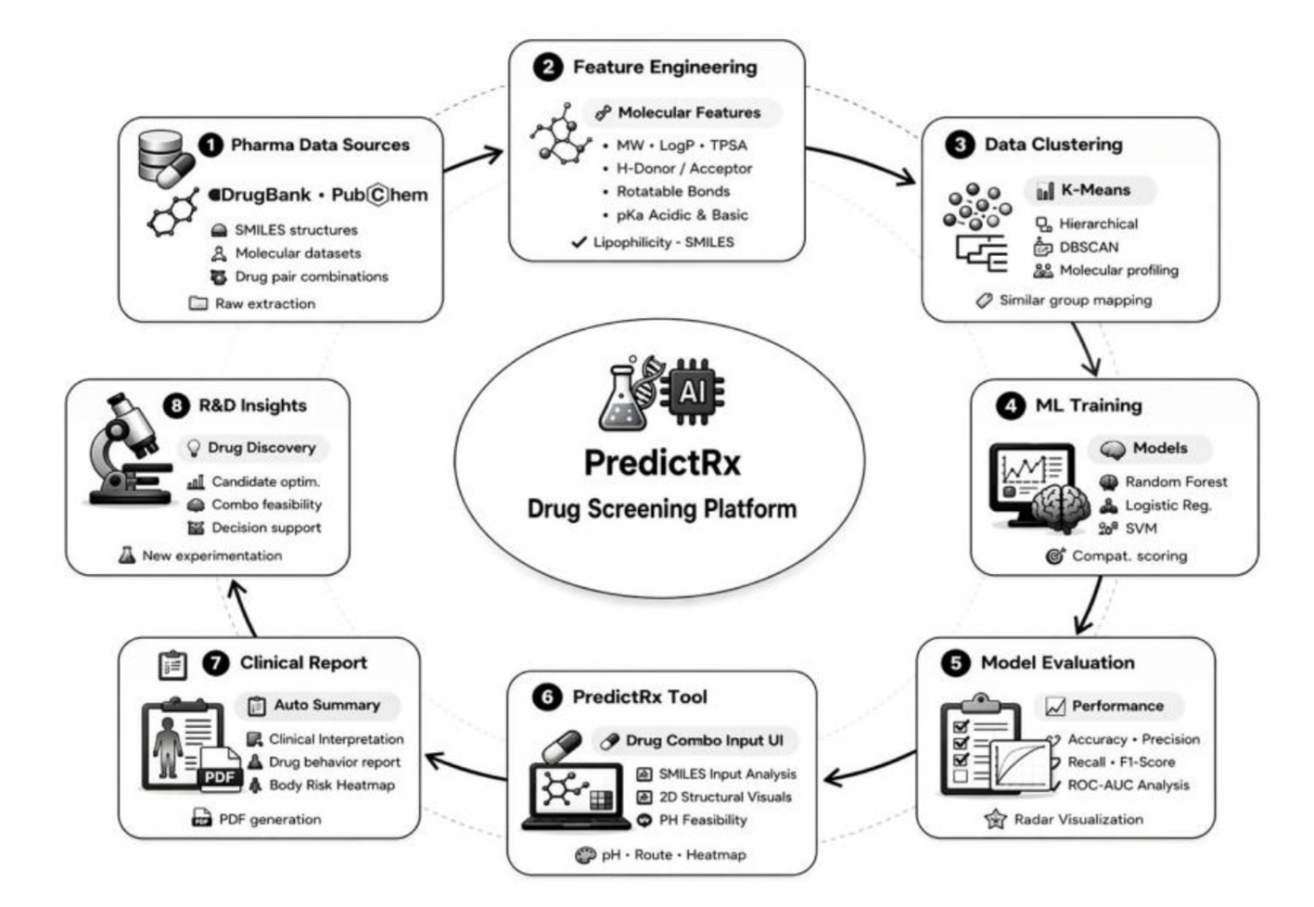
Illustration of the workflow of PredictRx.

PredictRx integrates molecular representation achieved through cheminformatics, assessments of physicochemical properties, and controlled machine learning on the identical tool of analysis. The tool employs different predictive models, K-Means Clustering, Hierarchical Clustering (Agglomerative) and DBSCAN were used as the clustering analysis methods to determine structural similarities, hierarchies, and density-based clustering of drug compounds and classification models were used to predict drug behavior and interactions based on molecular features. Three classifiers RF, SVM, and LR were used together for cross model comparison. Besides, PredictRx also incorporates drug combination analysis and structured visualization tool which allows the researcher to examine the potential interactions of synergy and compare candidate compounds given different pharmacological conditions. The overall workflow of the tool from data processing to prediction stages, is illustrated in Figure 1.

PredictRx will be incorporated to serve as an addition to the computational drug recommendation activities and aid in the development of the more efficient process of developing therapeutic strategies in the context of breast cancer by bringing together predictive modeling, protein structure prediction and interpretable decision support features.

1. The study focuses on the design and development of PredictRx, an artificial intelligence based molecular screening tool specifically tailored for breast cancer drug recommendation, enabling efficient analysis of drug interactions and candidate evaluation.
2. It involves the implementation and evaluation of various machine learning models to predict key physicochemical properties and drug likeness characteristics of candidate molecules, supporting data driven decision making in early-stage drug recommendation.
3. An integrated decision support framework is developed to assist in identifying, analyzing, and prioritizing potential drug candidates based on predictive insights generated by the tool.

## 2 Related work

One of the most serious global health challenges is breast cancer as, GLOBOCAN 2020 and estimated approximately 2.3 million newly diagnosed breast cancer patients worldwide (Lavecchia, 2015). At the same time, high failure rates, lengthy research and development process, and escalating costs of research and development define pharmaceutical drug recommendation pipeline (Schneider et al., 2020; Bender and Cortés-Ciriano, 2021; Qureshi et al., 2023). To address these issues, AI has been adopted in the pharmaceutical research process to accomplish tasks such as the screening of the molecules, the target, and predictive analysis (Vamathevan et al., 2019; Schneider et al., 2020; Bender and Cortés-Ciriano, 2021). Algorithms like RF, SVM, and LR have been widely applied in oncology related classification tasks (Zagidullin et al., 2019; Wang et al., 2023), and in the quantitative structure-activity relationship (QSAR) model to predict drug recommendation outcomes (Zagidullin et al., 2019; Wang et al., 2023).

The cases of deep learning and molecular representation learning have improved the prediction of molecular properties and compound activities by means of desktop models and molecular fingerprints (Chen et al., 2018; David et al., 2020; Feinberg et al., 2018; Zhou, 2021; Walters and Wang, 2020). In addition to this, there are several computational studies assessing drug combinations and assessing synergistic interactions, such as the DeepSynergy model of cancer drug combinations (Preuer et al., 2018; Liu et al., 2024; Pan et al., 2023; Zhao et al., 2025) and graph-based models (such as GraphSynergy) which use graph neural networks to predict synergy. In addition to this, more databases such as DrugComb have been developed to standardize experimental evidence on drug combinations and perform synergy research (Zagidullin et al., 2019; Esteva et al., 2019; Kim et al., 2019; Wishart et al., 2018), and other scholars focus on the growing importance of artificial intelligence-based decision-support tools in healthcare analytics and treatment planning (Chen et al., 2018; Topol, 2019; Hoghooghi Esfahani et al., 2025). Furthermore, it has demonstrated that AI-based virtual screening models would be successfully applicable to optimize the efficiency of the compound identification, in the early phases of candidate (Stokes et al., 2020; Qureshi et al., 2023).

The wider implications of artificial intelligence on the medical field and biomedical research have also been widely examined, and the interest was on AI's growing role in reshaping how clinical and biomedical decisions are made (Chen et al., 2018; Topol, 2019; Hoghooghi Esfahani et al., 2025).

The pharmaceutical industry is undergoing tremendous digital revolution strategically in order to boost productivity in the R&D through use of advanced computational tools and data-driven strategies (Zagidullin et al., 2019; Qureshi et al., 2023). Recent advancements in graph neural networks and explainable artificial intelligence have further improved drug interaction prediction and model interpretability (Gawehn et al., 2016; Ying et al., 2019; Rives et al., 2021). Most of the existing literature, however, is focused on either the prediction of molecular properties, diagnostic or to the analysis of drug combinations separately, with minimal combination into a coherent workflow of decision support. It is this literary gap that directs toward the need to have integrated AI-based frameworks that can bring molecular screening, predictive analysis, and drug synergy analysis in one framework to aid research and first drug recommendation in the field of oncology. The Table 1 is a compilation of related work performed previously.

**Table 1:** Previous literature work.

S. No	References	Category	Key contribution	Gap identified	How PredictRx addresses these
1	Chen et al., 2018	AI in drug recommendation	Demonstrates the use of deep learning for molecular representation and prediction	Focuses mainly on prediction, lacks integration with drug synergy analysis	PredictRx integrates molecular prediction with drug compatibility and synergy evaluation
2	Vamathevan et al., 2019	AI in pharmaceutical research	Comprehensive overview of machine learning applications across drug recommendation pipeline	Does not provide a unified system for end-to-end prediction	PredictRx offers an integrated decision-support framework combining multiple stages
3	Schneider et al., 2020	AI-driven drug design	Highlights AI-based approaches for drug design and optimization	Limited emphasis on real-time prediction and drug interaction modeling	PredictRx enables predictive screening and interaction-based analysis
4	Bender and Cortés-Ciriano, 2021	AI evaluation	Discusses realistic expectations and limitations of AI in drug recommendation	Lack of practical implementation frameworks	PredictRx provides a functional decision-support system
5	Lavecchia and Cerchia, 2019	Machine learning in drug recommendation	Explores ML techniques such as RF, SVM, and LR in drug recommendation	Does not include drug synergy or combination analysis	PredictRx incorporates synergy prediction along with ML models
6	Srivastava et al., 2020	AI applications in pharma	Reviews AI applications in pharmaceutical research and development	Broad and generalized without specific system implementation	PredictRx provides a targeted and applied framework
7	Preuer et al., 2018	Drug synergy	Introduces DeepSynergy model for predicting anticancer drug combinations	Focuses only on synergy without integrating molecular screening	PredictRx combines synergy prediction with molecular analysis
8	Zhang et al., 2021	Graph neural networks	Uses GNNs for drug synergy prediction	Limited dataset scope and lack of integration with other models	PredictRx uses diverse datasets and integrates multiple ML techniques
9	Zagidullin et al., 2019	Drug databases	Develops DrugComb database for drug combination analysis	Provides data but lacks predictive modeling capabilities	PredictRx builds predictive models on top of such datasets
10	Sutton et al., 2020	Clinical decision support	Highlights AI-based decision support systems in healthcare	Not specifically tailored for drug recommendation	PredictRx focuses on drug-specific decision support
11	David et al., 2020	Molecular representation	Discusses molecular descriptors and representation techniques	Does not integrate ML pipelines for prediction	PredictRx integrates descriptors with ML-based prediction models
12	Chen et al., 2018	Drug–target interaction	Explores in silico drug–target interaction prediction	Does not address drug synergy or combination effects	PredictRx extends prediction to drug compatibility and synergy
13	Wang et al., 2023	Machine learning in oncology	Reviews ML-based cancer prediction models	Focuses on diagnosis rather than drug recommendation	PredictRx focuses on therapeutic prediction and drug interactions
14	Qureshi et al., 2023	AI in drug recommendation	Discusses big data and AI integration in drug recommendation	Lacks implementation of a unified predictive system	PredictRx integrates data, models, and decision support into one framework
15	Walters and Murcko, 2020	Generative AI in chemistry	Explores generative AI in medicinal chemistry	Limited clinical or application-based integration	PredictRx bridges molecular generation with predictive analytics

## 3 Methodology

### 3.1 Overview of the tool and research design

**Figure 2:**
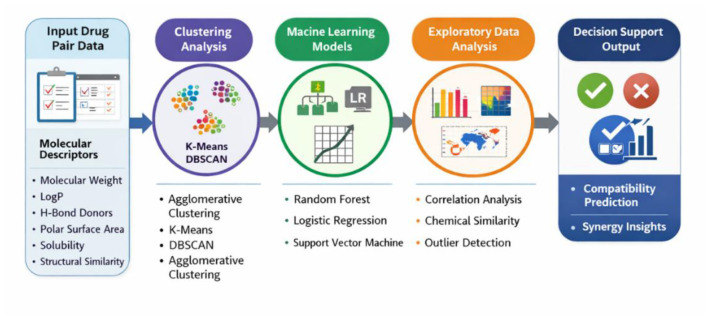
Illustration of step-by-step map of the drug input to compatibility prediction process.

The proposed tool is called PredictRx and is created as a web-based decision support tool that will analyze possible drug interactions and drug synergy patterns in case of breast cancer treatment. The tool integrates machine learning methods based on data analysis with pharmaceutical knowledge domain to facilitate the predictive analysis of drug compatibility. The general process of research includes data collection, dataset construction, preprocessing and feature engineering, model construction with unsupervised and supervised methods of learning, model testing with statistical measures, and deployment of the tool with the help of an interactive interface, this process is illustrated in Figure 2. The technical implementation will guarantee validity and long-term applicability of the PredictRx into the pharmaceutical and biotechnology sector.

### 3.2 Data collection and construction

The process of data collection began with finding breast cancer drugs on publicly accessible resource (List of 96 Breast Cancer Medications Compared, n.d.), a curated list of medications that are used in the process of treating breast cancer. Public databases (Kim et al., 2019) and DrugBank (Wishart et al., 2018) were then searched to obtain detailed information about the drugs. However, each database was identified to derive the relevant information breast cancer medication was retrieved through drug-drug interaction information (List of 96 Breast Cancer Medications Compared, n.d.). Further, the detailed pharmacological information was discovered with potential mechanism of action with relative target proteins and pathway annotations (Wishart et al., 2018). Also, the complete molecular structure was obtained with physicochemical descriptors (Kim et al., 2019). In addition, there were several experimental validated synergy data labels which was not available for all possible drug combinations. Hence, synergy labels were generated using a computational approach, which were assigned using a rule-based framework that combined information from documented interaction profiles, molecular similarity measures, target overlap, pathway associations, and complementary mechanisms of action.

Further analysis revealed that complete information was available for 81 drugs, which were subsequently included in the study. The databases contain credible chemical, biological and pharmacological data needed to do computing analysis. The attributes that are collected are considered fundamental like names, classes of various drugs, molecular properties, mechanism of action, target protein and receptors, biological processes that are involved in breast cancer and therapeutic implication in the practice of oncology. All of these properties create a complete image of a particular drug and form the basis of machine learning model formation. These databases are widely used in artificial intelligence driven drug recommendation for large scale molecular data integration and predictive modeling (Kim et al., 2019; Wishart et al., 2018; Qureshi et al., 2023).

After collecting the data, the received information will be grouped into a structured dataset that can be analyzed by machine learning. The drugs are represented by their attendant attributes and drug drug interactions are systematically generated to study the interaction patterns. Such combinations are possible therapeutic combinations that can have a complementary and/or synergistic effect. The dataset is built by combining drug features of individual drugs with combined features of drug pairs such as predicted or inferred interactions on the basis of molecular pathways and biological pathways. The resulting dataset is thus made up of individual drug attributes, pair-wise drug combinations and inferred interactions. The data that is used in predicting drug compatibility has a total of 3160 samples with 34 features, 2528 of which will be used in the training of the model and 632 samples in the testing of the model, guaranteeing a balanced and valid structure of model training and testing.

### 3.3 Optimization of data and feature engineering

Preprocessing and feature engineering methods are used before training a model in a bid to guarantee the quality and uniformity of data. This involves eliminating duplicate records, working on missing records and standardization of data. Moreover, to minimize the risk of data leakage, the dataset was divided into training and test sets before model development. All preprocessing steps were carried out using only the training data and then applied to the test set. In addition, incomplete and duplicate records were removed during data curation. The encoding techniques are used to transform categorical variables like drug class, type of mechanism, and type of therapy category into numbers to be used in machine learning algorithms. Moreover, feature engineering is also used to obtain new variables that reflect connections among molecular properties, biological pathways, and mechanisms of action. These artificial attributes increase the capacity of machine learning models to identify sophisticated trends related to drug interactions and synergy. The preprocessing pipeline is used to make sure that all of the input data has been converted to a standard format which can be used by the clustering and classification algorithms to improve the performance and reliability of the model.

### 3.4 Model development

PredictRx uses both unsupervised and supervised machine learning models to predict drug interaction patterns. During the unsupervised learning stage, hidden structures and relationships between drugs, based on molecular and biological properties, are identified using clustering algorithms. K means clusters drugs according to similarity and distance to cluster centroid enabling similar drugs to be clustered. DBSCAN identifies clusters using the density of data and also determines noise points and outliers in the dataset. A hierarchical approach, agglomerative clustering, combines similar data points into clusters progressively. These clustering methods assist in exposing the latent patterns in the data and give indications on similarities and grouping of drugs, and hence aid in the analysis of drug interactions (Feinberg et al., 2018; Gilmer et al., 2017; Zhou, 2021). After clustering, supervised learning models are used to forecast the outcomes of the drug interactions. Random Forest is an ensemble learning technique that integrates a series of decision trees to enhance the precision of prediction and lower the variance as well as minimize overfitting. Logistic Regression is a statistical model to approximate the likelihood of the interaction outcomes, depending on the input features. Support Vector Machine (SVM) finds the best decision line to determine compatible and incompatible drug pairs. They are trained with processed datasets and applied to the prediction of the possible synergy and compatibility between drug combinations, which have shown good performance in biomedical prediction problems and drug recommendation (Lavecchia, 2015; Bhatt et al., 2024; Qureshi et al., 2023). Furthermore, hyperparameter tuning was accomplished via grid-searching to improve model performance for all supervised learning algorithms. Random forest parameters included number of trees, maximum depth of trees and minimum number of samples to split a node. Support vector machines hyperparameter optimization searched for kernel type, regularization parameter (C) and gamma values. Similarly, logistic regression hyperparameters were evaluated with regularization strength and penalty functions. The best performing hyperparameter combinations were then selected to fit the final models.

### 3.5 Model verification and evaluation

To make sure that the machine learning models developed in terms of the PredictRx are reliable, accurate, and effective, the results of the developed models are carried out with the help of a set of the validation metrics. In the case of clustering models they use internal validation methods to evaluate the quality, unity and isolation of clusters. The Silhouette Score is one of the main tools applied, and it is based on the fit of a particular data point to the given cluster in contrast to other clusters. It contains intra cluster similarity and inter cluster dissimilarity, a value of +1 indicates clearly defined clusters. The Silhouette coefficient of a given data point is represented as:


$$\begin{array}{l} S\left(i\right)=\frac{b\left(i\right)-a\left(i\right)}{\max\left(a\left(i\right),b\left(i\right)\right)} \end{array}$$ (1)

Here, a(i) measures average distance between data points intra cluster wise in the same cluster, and b(i) captures the lowest average distance between data points and other data points in other clusters.

The Calinski-Harabasz Index is also another significant measure of clustering that measures how well the between-cluster dispersion is compared to the within-cluster dispersion. When this index has higher values, then more definite and clear clusters are found, which implies that the clustering structure is reasonable. This is calculated by the following equation:


$$\begin{array}{l} CH=\frac{Tr\left(B_{k}\right)}{Tr\left(W_{k}\right)}\times \frac{n-k}{k-1} \end{array}$$ (2)

where Tr(Bk) represents the between-cluster dispersion, Tr(Wk) represents the within-cluster dispersion, nis the total number of observations and k is the number of clusters.

Lastly, the Davies-Bouldin Index is employed to quantify the mean distance between clusters, considering intra-cluster distance and the distance between cluster center. The smaller the values of this index are, the more performance in clustering, the less similarity there is between clusters and the more distinctiveness. The index is given by:


$$\begin{array}{l} DB=\frac{1}{k}\overset{k}{\underset{i=1}{\sum }}\max_{j\neq i}\left(\frac{S_{i}+S_{j}}{M_{ij}}\right) \end{array}$$ (3)

where Si and Sj represent intra-cluster distances and Mij represents the distance between cluster centers.

In the case of classification models, a number of standard metrics are used to determine performance, and they reflect various aspects of predictive accuracy. The measure of Accuracy gives the general idea of the correctness of the model and is the total percentage of correct classifications relative to the number of observations. It is calculated as:


$$\begin{array}{l} Accuracy=\frac{TP+TN}{TP+TN+FP+FN} \end{array}$$ (4)

TP, TN, FP and FN are the true positives, true negatives, false positives and false negatives, respectively.

Another important metric is Precision; that measures the reliability of positive predictions is the extent to which positive predictions are correctly predicted. It is expressed as:


$$\begin{array}{l} Precision=\frac{TP}{TP+FP} \end{array}$$ (5)

Recall is the response to the question of how well the model is able to recognize real positive cases thus showing how the model is able to capture the relevant cases. It is defined as:


$$\begin{array}{l} Recall=\frac{TP}{TP+FN} \end{array}$$ (6)

To give a balanced analysis, particularly where there are imbalanced datasets, F1-score is taken as the harmonic mean of precision and recall. This measure will make sure that the false positives and the false negatives are taken into account at the same time. It is given by:


$$\begin{array}{l} F1=\frac{2\times \left(Precision\times Recall\right)}{Precision+Recall} \end{array}$$ (7)

Besides these measures, a visual tool of the confusion matrix is also used to compare the real and the expected classifications where it gives an indication of the presence of the true positives, true negatives, false positives, and false negatives.

Moreover, the Receiver Operating Characteristic (ROC) curve is used to analyze the model performance with different classification thresholds by plotting the rate of true positives vs. false positives and any of these are calculated as:


$$\begin{array}{l} TPR=\frac{TP}{TP+FN},FPR=\frac{FP}{FP+TN} \end{array}$$ (8)

The general performance of the classifier is also summed up with the help of the Area Under the Curve (AUC) that indicates the capacity of the model to differentiate between classes. The greater the AUC, the better is the discriminative performance. The AUC is defined as:


$$\begin{array}{l} AUC=\int_{0}^{1}TPR\left(FPR\right)d\left(FPR\right) \end{array}$$ (9)

All these assessment measures give a powerful and holistic analysis of clustering and classification models in the tool that guarantees that the constructed models are correct, dependable and ideal to be used in predictive analysis.

### 3.6 PredictRx architecture and operating

PredictRx tool starts with user input where the user selects drug combinations, then these inputs are mapped to the appropriate data features including molecular properties, mechanisms of action, and biological pathways of every drug. This input is fed into the tool by a preprocessing pipeline which makes sure that the data is encoded, standardized, and transformed in a way that is consistent with the format used during training then forwarded to the predictive architecture and the model makes an inference to identify relationships within the set of drugs. The models assess the potential of interaction or synergy generated with the help of learned features and provide predictions. The presentation of the results is done in a clear and interpretable format through the user interface, as well as explanatory insights that are used to make the user comprehend the reasoning behind the forecast. The tool is founded on a modular architecture which can be expanded in future with more datasets, models, and analytical features. It is a workflow that is efficient and scalable to support decision making in breast cancer research on drug interaction analysis.

### 3.7 Development of “PredictRx” decision-support tool

PredictRx is created as the web tool that combines the most efficient machine learning model that has been found throughout the assessment. This tool was developed in Python as the main programming language and deployed via streamlit, allowing access to the tool with a web browser (PredictRx WebTool, n.d.). The tool provides user a possibility to interact and visualize the results in real time. The user chooses the combinations of drugs and makes predictions on how they interact or synergistically react. PredictRx is user friendly and easy to use by researchers in the healthcare field. The tool has both qualitative predictions and explanatory ideas based on the outputs of the models that users can make use of to investigate drug interactions.

## 4 Results and discussion

### 4.1 PredictRx output and analytical results

The current paper assesses the functionality and the usability of the PredictRx AI based drug screening decision support tool to predict drug compatibility and synergy. The system combines machine learning algorithms and molecular descriptor-based analysis of drug pair data. Physicochemical properties that are used to describe each drug pair include molecular weight, lipophilicity (LogP), hydrogen bond donors and acceptors, polar surface area, solubility and structural similarity indices. Such descriptors play a critical role in defining pharmacokinetic activity, the potential of such interplay, and the general compatibility of therapy. PredictRx also conducts three primary analytical tasks, namely, clustering, supervised classification and exploratory data analysis (EDA) that can be combined to get a thorough insight into the dataset. Clustering allows identifying the natural chemical groupings, models of classification give predictions of the result of compatibility, and EDA gives statistical and graphical information about the relationship between molecules. The combination of these modules is in such a way that the tool does not only predict but it elucidates the underlying patterns to improve the level of interpretability (Stokes et al., 2020; Hu et al., 2018).

In a larger sense, the analytical results of PredictRx can be used as a first line screening tool in drug recommendation pipelines. The tool helps to cut down time and cost of the traditional laboratory-based screening by determining compatibility patterns and synergistic potential before experiments can be undertaken to validate them.

### 4.2 Clustering model results

The clustering analysis was conducted to determine the inherent form of the data as well as to discover clusters of drugs with common molecular properties. Three unsupervised learning algorithms, which are K-Means, DBSCAN, and Agglomerative Clustering were applied and tested with three internal metrics Silhouette score, CH Index and DB Index. All these metrics measure cluster cohesion, separation and the quality.

It can be seen that Agglomerative Clustering scored better than the other algorithms achieving the highest Silhouette score (0.6946) and the least DB Index (0.2457), both indicating strong cluster separation. K-Means had moderately good clustering, which implies that although it can find broad clusters, it might not be able to effectively find detailed hierarchical relationships in the data. DBSCAN was the most underperforming as it is subject to changes in density and probably led to a collection of fragments or irregular clusters, all three clustering metrics are summarized in Table 2.

**Table 2:** Comparison of the clustering algorithms.

S. No.	Model	Silhouette score	Calinski-Harabasz index	Davies-Bouldin index
1	K-means	0.4287	52.1569	1.0085
2	DBSCAN	0.2973	17.2197	2.442
3	Agglomerative	0.6946	34.8422	0.2457

**Figure 3:**
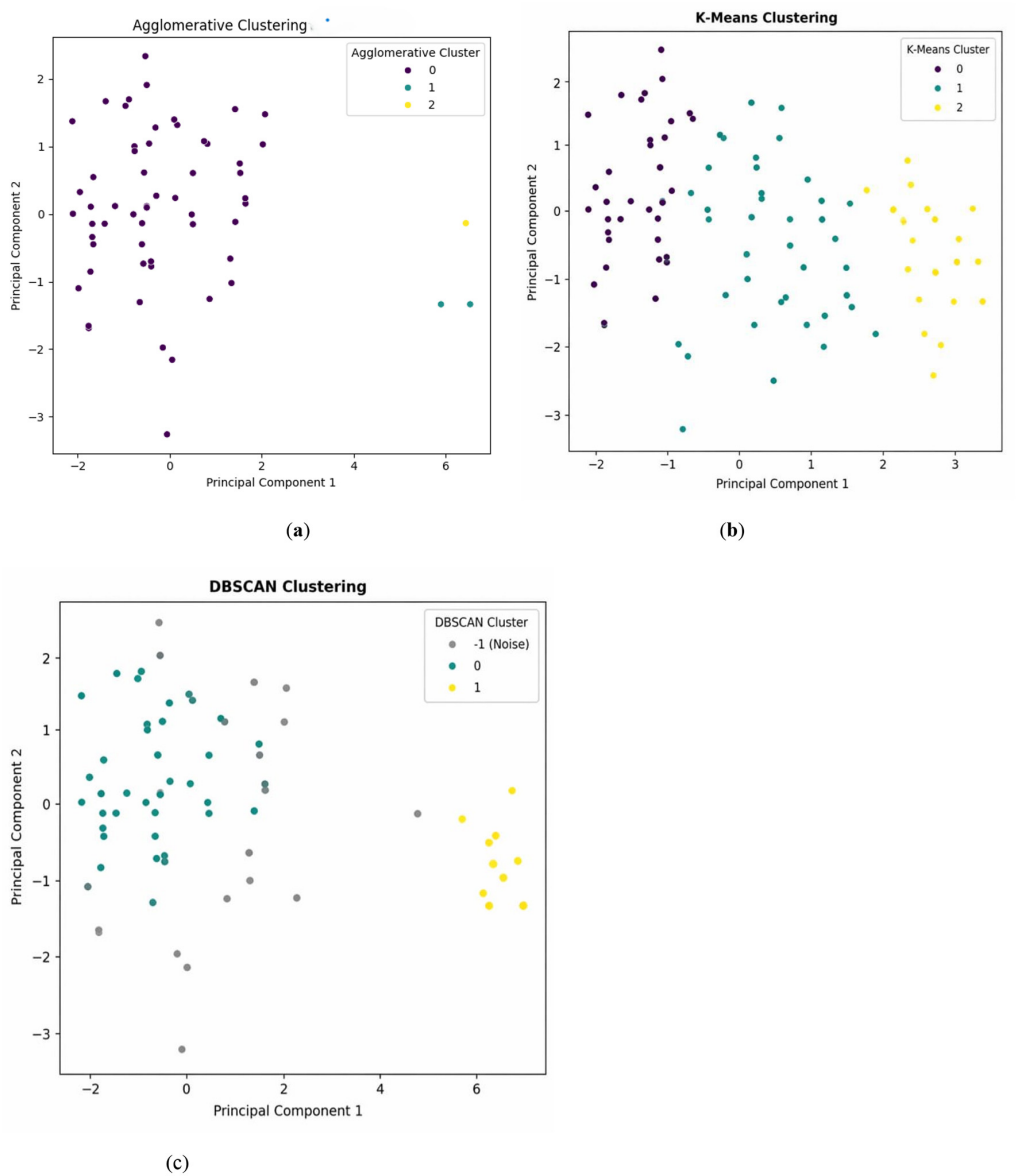
Illustration of PCA for different clustering model. **(a)** Visualization for Agglomerative Clustering. **(b)** Represents the K means Clustering. **(c)** Shows DBSCAN clustering.

Clustering outcomes were also graphically represented with the help of Principal Component Analysis (PCA) that compressed high-dimensional molecular descriptors space in two major components. The Figure 3 showed (A) Agglomerative clustering, (B) DBSCAN clustering and (C) K-means clustering, which explains that there exist three different clusters in each model. These clusters are grouped into similar drugs in terms of physicochemical properties cluster naturally. These clusters may be viewed as the expression of various chemical classes or pharmacological action.

In drug recommendation, this type of clustering can be of great value as it allows finding structurally or functionally similar compounds which can share biological behavior. Also, clustering may help in the process of eliminating candidate drugs to use in combination therapy by clustering compatible compounds. This fact is supported by the better performance of Agglomerative Clustering, which implies that hierarchical relationships between the drug features are important to determine compatibility patterns.

### 4.3 Results of model verification and validation

Random Forest, Logistic Regression, and Support Vector machine were three supervised machine learning models whose performance was predicted to assess the predictive power of the PredictRx tool. Standard metrics including accuracy, precision, recall, F1 score and AUC-ROC combined were used to evaluate these models.

Random Forest was the best performing model among those that were assessed and its metrics is 1.00, which is a good predictor. Logistic Regression and Support Vector Machine also performed well with the accuracy of 0.909 and were a little lower than the accuracy of Random Forest, which can be observed in Table 3. The high performance of the Random Forest can be explained by the fact that the algorithm is based on ensemble learning where a number of decision trees are combined to both utilize complex, non-linear relationships in data as well as reduce overfitting.

**Table 3:** Performance metrics table.

S. No.	Model	Accuracy	Precision	Recall	F1 score	ROC AUC
1	Random forest	1.00	1.00	1.00	1.00	1.00
2	Logistic regression	0.909	0.910	0.909	0.906	0.963
3	Support vector machine	0.909	0.911	0.909	0.909	0.975

**Figure 4:**
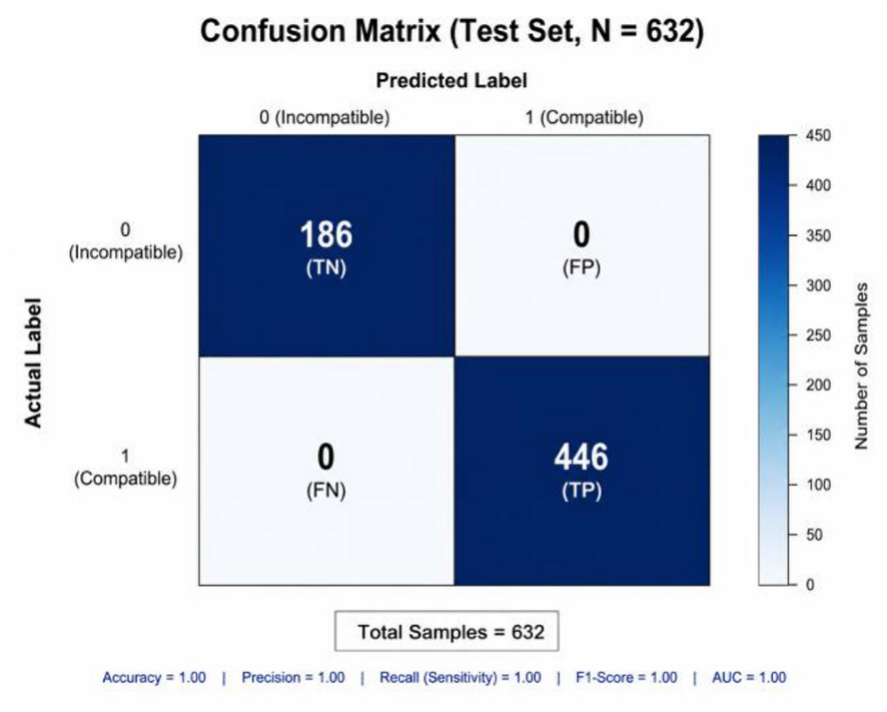
Illustration of confusion matrix.

The confusion matrix illustrated in Figure 4 also confirms the usefulness of the Random Forest model. All validation samples were correctly classified with no false positives or negatives recorded. This implies that the model has been able to learn the relationship between molecular descriptors and the outcome of drug compatibility without any classification error.

In its practical application, the lack of false positives and false negatives is of paramount importance as a wrong classification in the prediction of drug compatibility may result into the ineffective or even detrimental combinations of drugs. Thus, the recorded performance is indicative of the strength and stability of the model to facilitate the decision-making processes.

#### 4.3.1 ROC curve analysis

**Figure 5:**
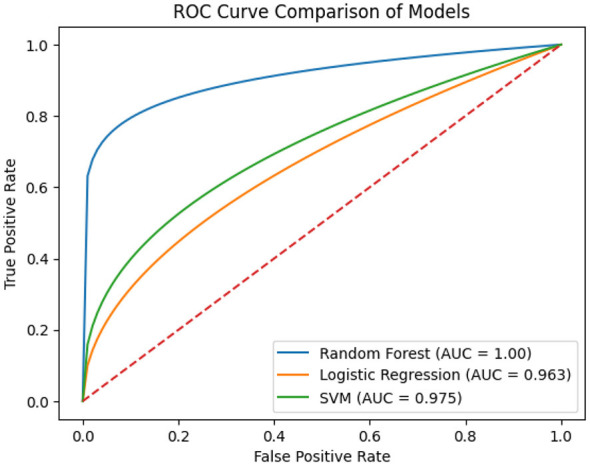
Illustration of ROC curve.

The analysis of the ROC curve also confirms the discriminative ability of the model and Random Forest achieved a perfect AUC of 1.00 on the ROC curve, while SVM and LR were outperformed by RF, which is illustrated in Figure 5. It implies that the separation of compatible and incompatible drug pairs is perfect at all classification thresholds. This performance is an indicator of the generality quality of the model and the stability of prediction accuracy. It should be noted though that extremely high performance can also be indicative of possible overfitting in situations where relatively small datasets are used. Consequently, its generalizability is suggested to be validated with additional data sources in order to establish the validity of the model.

### 4.4 Exploratory data analysis (EDA)

Exploratory Data Analysis was carried out to analyze the statistical characteristics, distribution patterns and interrelationships of the molecular descriptors of the dataset. The data set has 3160 records of drug pairs with 34 molecular descriptors and a small percentage of missing values (2.5), which is an indication of good data and good use in the machine learning analysis process. Minimal missing values also decrease the level of imputation required and guarantee the dependability of the patterns observed and their reliability to the dataset.

EDA is important in revealing the effects of the molecular properties on the drug compatibility because it can reveal concealed trends that can never be clearly identified through predictive models. Through the distribution and correlation analysis and structural similarities, an interpretation of the role of various physicochemical attributes in the compatibility results is achievable. This is especially necessary in drug use where predictability must be able to be interpreted and reasoned scientifically.

#### 4.4.1 Molecular weight distribution

The molecular weight distribution is determined by the balance between molecular mass and concentration of amino acid in the solution. The analysis of the molecular weights distribution shows that compounds have most of their concentration at 512.91 g/mol. This fact coincides well with the Rule of Five proposed by Lipinski, which indicates that molecules that have drug-like properties are usually characterized by molecular weight less than 500–600 g/mol to guarantee the presence of the sufficient bioavailability. This extension of the limit to 500 g/mol by a small margin shows that moderately complex compounds are probably present in this data, and they can be pharmacologically active.

**Figure 6:**
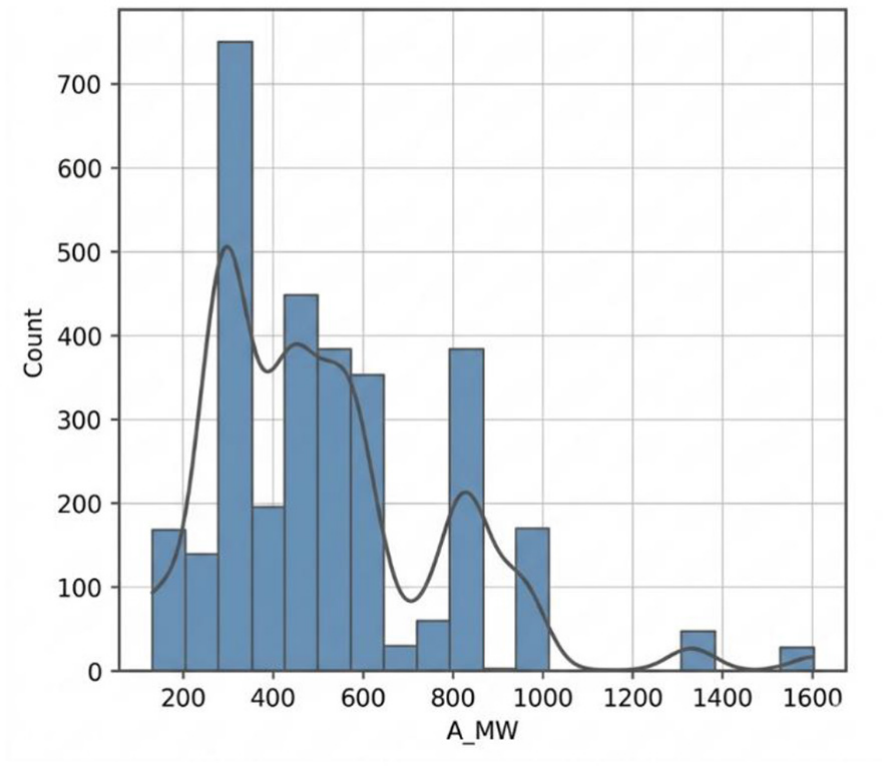
Drug A molecular weight values.

Analytically, this distribution validates that the data is made up of therapeutically relevant structures and not random chemical structures. This is significant in that machine learning models that have been trained on compounds with drug-like properties stand better chances of making clinically significant predictions. Also, a comparatively low dispersion of the molecular weight values indicates a consistency in the data, which means that there is less variability, which affects the model performance adversely. Advancements in computational drug recommendation have further expanded the interpretation of drug likeness and molecular efficiency, improving prediction accuracy in AI based tools (Hu et al., 2018). The histogram in Figure 6 illustrates that majority of the Drug A molecular weight values lie between 200–500 g/mol with few outliers having molecular weight more than 700 g/mol, which is structural diversity.

#### 4.4.2 Lipophilicity (LogP) profile distribution

The balance distribution of lipophilicity (LogP) has the maximum value of 2.32, which implies that the majority of compounds have the optimal hydrophobicity. Lipophilicity is also a very important determinant of drug behavior because it determines the membrane permeability, solubility, and absorption. A balanced LogP value would assure that the compounds can easily cross the biological membranes and also be soluble enough in water.

**Figure 7:**
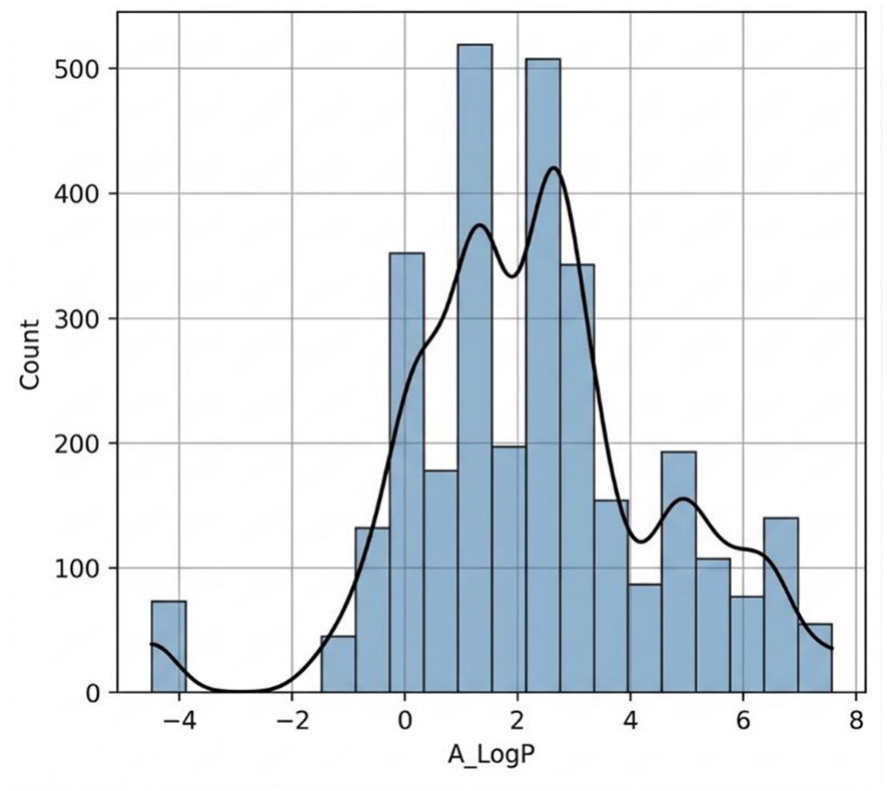
Drug A LogP values.

The distribution observed indicates that the dataset has compounds with good pharmacokinetic properties and hence it is appropriate in the analysis of compatibility prediction. Moreover, the lack of extreme values of LogP assumes that the dataset does not include highly hydrophobic or highly hydrophilic compounds, both of which can cause a poor performance of the drug. This equilibrium makes the predictive modeling more reliable because extreme values are a major contributor to noise and lower generalizability of the model. The histogram illustrated in Figure 7 indicates that most of the Drug A LogP values are centered around 0–3 with some outliers at 5, which indicates that drugs A differ in their lipophilicity.

#### 4.4.3 Drug interaction heatmap analysis

The heatmap analysis would give an understanding of the correlations of differences in molecular descriptors with drug compatibility. It was found that the positive correlations existed between features like differences in rotatable bonds, TPSA, MW, HBA and HBD. These findings demonstrate that drugs that have minor variations tend to be more effective in combination since they complement each other at the molecular level.

**Figure 8:**
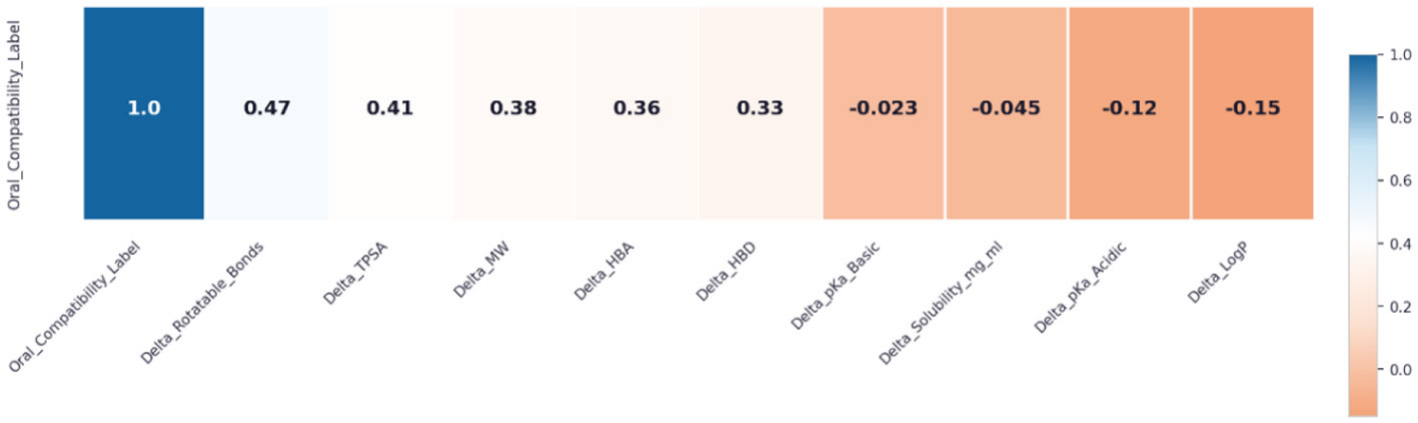
Correlation of molecular features with oral compatibility.

Conversely, differences in acidity (pKa) and lipophilicity (LogP) were found to be negatively correlated meaning that extreme changes in these attributes can decrease compatibility. This is attributable to the fact that a large change in chemical properties can cause instability of the properties, decreased binding efficiency, or poor interaction between the drug molecules. In that the heatmap analysis is indicative of the fact that compatibility is not merely based on similarity but on a moderate association of similarity and differences. These results are significant as they assist us in selecting the combination of drugs to use. When we know the differences between the compounds, we can select the most appropriate pair to enhance the effectiveness of a medicine. According to the heatmap in Figure 8, the features that are moderately positively correlated with oral compatibility are rotatable bonds (0.47), TPSA (0.41), and molecular weight (0.38), and the features with weak or negative correlation are those of LogP (−0.14) and pKa (−0.03).

#### 4.4.4 Chemical distance and similarity analysis

The correlation between structural similarity and drug compatibility is also supported by the evaluation of chemical distance and Tanimoto similarity. The findings show that a pair of drugs are usually similar in chemical distance and structure when they are compatible as opposed to when they are not. This implies that structurally related compounds have higher chances of interacting in a synergistic manner.

**Figure 9:**
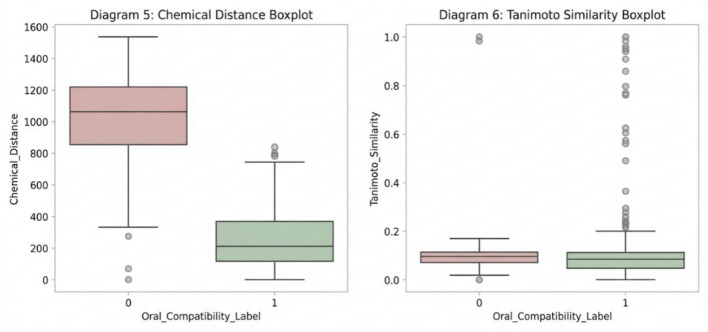
Compatible drug pairs.

It should be noted, however, that total similarity is not necessarily a good thing, since identical compounds do not necessarily have the extra therapeutic value when used together. Rather, the optimum degree of similarity pre-emits drugs to complement one another and yet retain specific functional functions. This strike between sameness and dissimilarity is one of the important points that can be used in the development of combination therapies. The results of this review support the value of molecular structure in the process of drug compatibility determination and underline the value of similarity-based measures in the context of predictive modeling. The boxplot in Figure 9 indicates that, compatible drug pairs are found to be closer in terms of chemical distance (0.2–0.4) and Tanimoto similarity (0.6–0.8), whereas non-compatible pairs are further apart in terms of chemical distance (0.6–0.8) and Tanimoto similarity (0.2–0.4), which implies that there are structural differences.

#### 4.4.5 Correlation matrix of global features

The worldwide correlation matrix shows that some molecular descriptors correlate very well with each other and in this case, the molecular weight, rotatable bonds, and polar surface area are associated with each other. Such correlations point out that some features could also be redundant and therefore could affect model performance by creating multicollinearity.

**Figure 10:**
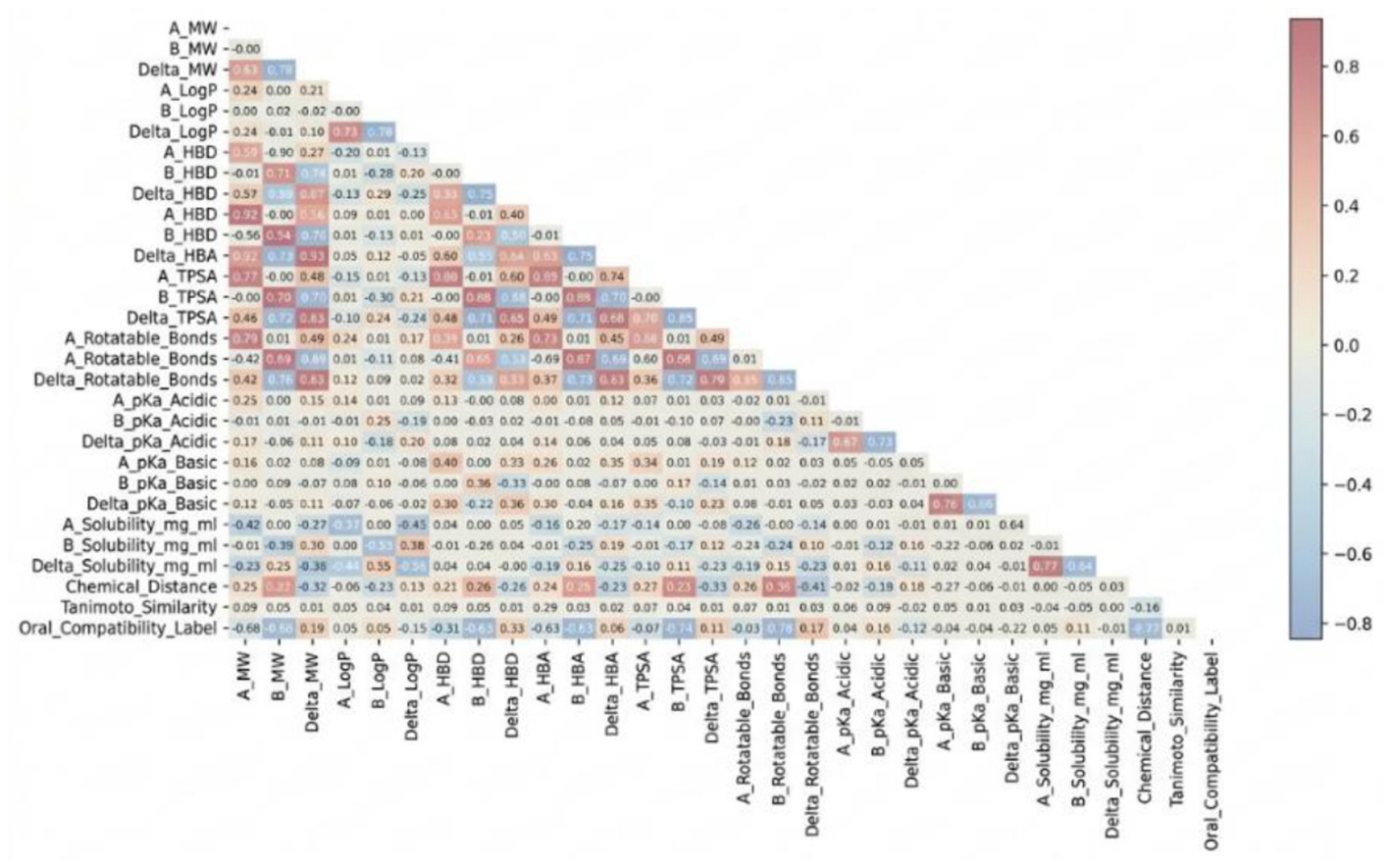
Correlation patterns among molecular features.

The existence of correlated features in a modeling point of view implies that dimensionality reduction or feature selection methods need to be applied in order to enhance efficiency and interpretability. Redundancy minimization can improve the performance of the models by maximizing on the most informative features and reducing the noise. Also, the correlation matrix offers useful information on the structural dependencies of the molecular descriptors, and can be used in the future to inform feature engineering in drug recommendation studies. The correlation matrix in Figure 10 demonstrates that a number of molecular features have moderate and strong correlations one of these correlations being that of molecular weight and TPSA (0.6–0.8), and others −0.2 to 0.2– are weak or negative, which means that some features are more dependent than others.

#### 4.4.6 Outlier detection analysis

The outlier detection analysis revealed few compounds that had extreme values in their molecular weight and lipophilicity. Although most of the dataset is consistent and homogeneous in nature, these outliers are compounds that are structurally unique that could respond differently in the biological system.

**Figure 11:**
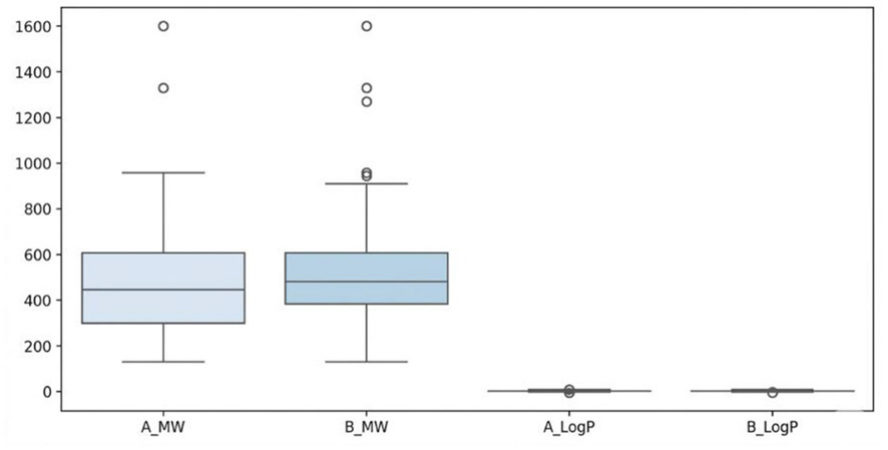
Distribution and variability of molecular features.

Analytically, outliers may have negative and positive sense. They can bring noise and decrease model accuracy on the one hand, or they can be new or unconventional compounds that have the potential to have therapeutic value on the other hand. Thus, one should not completely eliminate outliers instead of investigating them properly and learning how they affect model forecasts. The presence of outliers also speaks of the strength of the dataset as it contains the whole diversity of chemicals, but the entire dataset is consistent. The boxplot in Figure 11 depicts that there are outliers in the molecular features and the greatest numbers are within the interquartile range, and some of the extremes are beyond the whiskers, which is an indication of variability in molecular weight (200–600 g/mol) and LogP (0–4).

### 4.5 Quality and model robustness analysis

**Figure 12:**
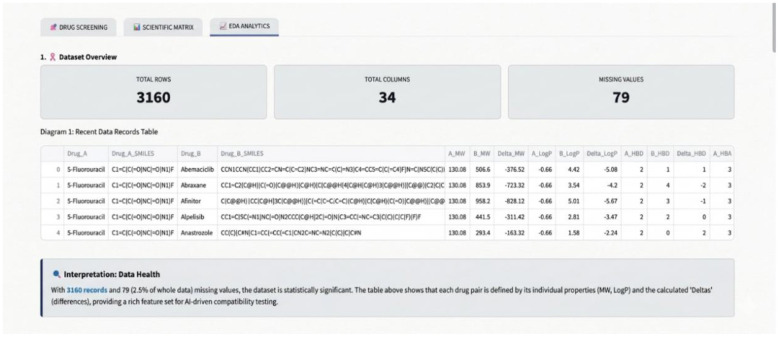
PredictRx EDA.

The robustness and the quality of the PredictRx was measured using the data integrity and the model performance. The data is of high quality having 3160 records, 34 features, 79 (2.5%) missing values and balanced distribution across molecular descriptors, which can be seen in Figure 12. This makes sure that the model is trained on quality and representative data and there is less chance of biased predictions.

Random Forest model has shown perfect classification performance which means high robustness and capability to capture complex relationships in the data. Nevertheless, this high performance can also indicate the possibility of overfitting especially considering the relatively small size of the validation set. This exhibits the need to test the model on external data to facilitate external validity.

Also, the outliers and the variability of some features can suggest that model predictions can be affected by extreme values. Thus, future iterations should employ cross validation, regularization and external data set benchmarking. All in all, the results have proven that, although the PredictRx is very precise, generalizability and real-life applicability is something one should think a lot before using it.

### 4.6 PredictRx interface

**Figure 13:**
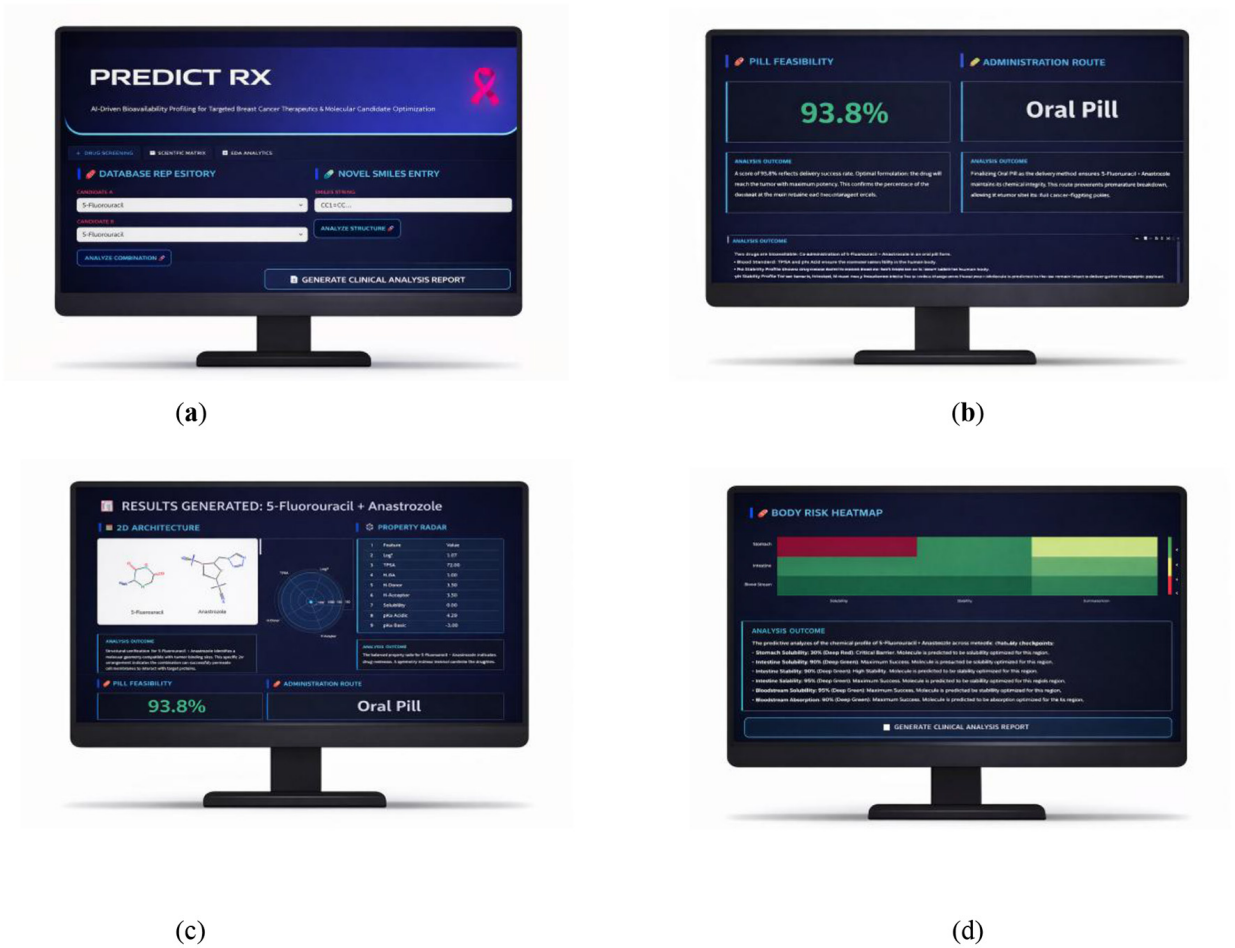
Various displays of the tool. **(a)** Represents the overall data repository. **(b)** Illustrates the efficacy of pill. **(c)** Demonstrates Pill Structure. **(d)** Overall effect of pill.

PredictRx is created as decision support tool which integrates certain analytical modules into one tool. The tool enables users to enter the data on drug pairings and receive the prediction on compatibility and synergy plus supporting analytical information. The interface is designed to show results in a readable and interpretable way such as clustering of outputs, classification results, and exploratory data visualizations. The main advantage of the interface is based on the possibility to combine several machine learning models to conduct comparative assessment and increase the accuracy of predictions. Such a multi-model method gives users the chance to know the strengths and weaknesses of each algorithm and this way enhance decision making. Moreover, the incorporation of visualization using heatmaps, distribution plots, and similarity measures increase the interpretation capabilities, and thus the tool can be used among technical and non-technical users. The interface design does not hamper the effective execution of workflow (input of data to interpretation of results) making it applicable in the research and preliminary screening application. Realistically, the PredictRx interface is a move toward making AI-based drug screening functional to researchers and pharmaceutical developers. Figure 13 illustrates various displays of the PredictRx tool that depeicts the results of analysis and prediction of drugs. Here, (A) Drug input interface, (B) Pill feasibility and Route of administration output, (C) Visualization of molecular analysis and (D) Visualization of heatmap of body risk.

### 4.7 Clinical implications

The findings of the PredictRx tool reveal that it has great possibilities to be used in the domain of drug recommendation in the early stages and in the study of combination therapies. The high predictive accuracy of the Random Forest model implies that machine learning might be successfully used to describe complicated associations between molecular descriptors and drug compatibility. This implies that PredictRx may be used as an initial screening tool, which allows researchers to establish which combinations of drugs may prove effective prior to experimental validation, which is costly and time intensive.

The clustering analysis, in addition, makes the tool more practical as structurally similar drugs will be grouped together, which may help locate compounds with similar pharmacological properties. These combinations give scientific rationale in the choice of candidate drugs to be used in combination therapies especially in situations where complementary mechanisms of action are needed. Moreover, the analysis of the exploratory data also underlines that molecular properties (lipophilicity, molecular weight, and similarity of structures) play an imperative role in establishing compatibility, and these data can be used in rational drug design.

Linkage of several analyses modules in one specific tool enhances efficiency through the facilitation of evaluation process. In general, PredictRx is an example of a decision-support tool that lies in the space between computational analysis and pharmaceutical research, and could be applied in drug screening as well as in future customized medicine methods.

### 4.8 Ethical considerations

The study is undertaken based on scientific data that are publicly available and does not entail patient level clinical data or personal health data. PredictRx tool is not a clinical diagnosis system or treatment recommendation; it is meant to be used only in academics and research. Results obtained by the tool are to be viewed as initial indications that need to be proved by experiment or clinical research before they can be put into practical use.

### 4.9 Future work

The next phase of the future development of the PredictRx tool is to enhance scalability, generalizability, and practical application. The increase in the dataset to the inclusion of the broader range of drug compounds is one of the main priorities as it will increase the strength of the model and improve its functioning in the context of different chemical structures. It is also necessary to validate the predictive models on independent datasets to determine whether the predictive models can be trusted or not in the context of the present dataset. The improvement of the model can be done through experimenting with new machine learning and deep learning methods, such as ensemble, XGBoost, LightGBM, graph neural networks, and hybrid models to better capture higher-order biological relationships and further improve model generalizability. Performance will also be optimized by incorporating the feature selection and dimensionality reduction techniques to eliminate redundancy in the molecular descriptors. Also, the combination of biological and pharmacological data, including protein-protein interactions or pathway data, may contribute substantially to better prediction and allow a much better insight into drug compatibility.

System-wise, creating a more interactive and easier-to-use interface will enhance usability to researchers. Reactivity and multiple user interface can be achieved through cloud deployment and API integration and make the tool more useful in practice. Ultimately, by ensuring that PredictRx is applied to distinct fields, including oncology and combination therapy studies, one will be able to make it even more relevant and help it to turn into a research prototype that can be later scaled to include drug recommendation. Furthermore, we will be incorporating more rigorous validation strategies, including stratified *k*-fold cross-validation, external validation using independent datasets, and prospective evaluation on newly generated drug combinations. These efforts will help further assess the robustness, generalizability, and real-world applicability of the proposed framework.

## Conclusion

This paper conducted research to understand how artificial intelligence has a possible impact in aiding in the drugs discovery to treat breast cancer. The conventional pharmaceutical development is costly, lengthy, and linked to high failure rates, which generate the necessity of computer tools that may assist in enhancing the screening and decision-making process at the early stages. As a solution to this problem, PredictRx was created, which is an artificial intelligence-based decision support tool, used in molecular screening and drug combination analysis.

PredictRx combines physicochemical property analysis, molecular representation which is based on cheminformatics, and supervised machine learning algorithms into a single analysis pipeline. Predictive performance was assessed using models like RF, SVM and LR classifiers and structural similarities between drug candidates were identified using clustering methods (K-Means Clustering, Hierarchical Clustering (Agglomerative) and DBSCAN). The addition of clustering methods also added more analytical power to the tool as it is now possible to identify structurally similar groups of drugs, this facilitates easier interpretation of drug combination and drug compatibility strategies.

The use of clustering and classification systems allowed the tool to identify a pattern in molecular data and help in anticipating possible drug interactions and synergistic effects. The tool was additionally implemented on basis of Python and web interface that facilitated real time interaction and result visualization. The findings indicate that machine learning methods can be used to promote computational drug screening, that is, to identify potential drug candidates, and drug combinations. The further comparison with the current AI tools revealed the possibility of aligning PredictRx as a decision-supporting instrument in AI based drug recommendation.

The future research can be aimed at using bigger biological samples, applying more sophisticated methods of deep learning, and creating a web based tool to conduct the analysis in real time. The practical use of PredictRx framework in the process of pharmaceutical research may be reinforced through the application of additional tools.

## Data Availability

The original contributions presented in the study are included in the article/supplementary material, further inquiries can be directed to the corresponding author/s.

## References

[B1] Altae-Tran H. Ramsundar B. Pappu A. S. Pande V. (2017). Low data drug recommendation with one-shot learning. ACS Cent. Sci. 3, 283–293. doi: 10.1021/acscentsci.6b00367 28470045 PMC5408335

[B2] Batool M. Ahmad B. Choi S. (2019). A structure-based drug recommendation paradigm. Int. J. Mol. Sci. 20, 2783. doi: 10.3390/ijms20112783 PMC6601033 31174387

[B3] Bender A. Cortés-Ciriano I. (2021). Artificial intelligence in drug recommendation: what is realistic, what are illusions? Part 1: ways to make an impact, and why we are not there yet. Drug Discov. Today. 26, 511–524. doi: 10.1016/j.drudis.2020.12.009 33346134

[B4] Bhatt P. Singh S. Kumar V. Nagarajan K. Mishra S. K. Dixit P. K. . (2024). Artificial intelligence in pharmaceutical industry: revolutionizing drug development and delivery. Curr. Artif. Intell. 2:E051223224198. doi: 10.2174/0129503752250813231124092946

[B5] Brown N. Fiscato M. Segler M. H. S. Vaucher A. C. (2019). GuacaMol: benchmarking models for de novo molecular design. J. Chem. Inf. Model. 59, 1096–1108. doi: 10.1021/acs.jcim.8b00839 30887799

[B6] Chen H. Engkvist O. Wang Y. Olivecrona M. Blaschke T. (2018). The rise of deep learning in drug recommendation. Drug Discov. Today. 23, 1241–1250. doi: 10.1016/j.drudis.2018.01.039 29366762

[B7] Chen R. Liu X. Jin S. Lin J. Liu J. (2018). Machine learning for drug-target interaction prediction. Molecules. 23:2208. doi: 10.3390/molecules23092208 30200333 PMC6225477

[B8] David L. Thakkar A. Mercado R. Engkvist O. (2020). Molecular representations in AI-driven drug recommendation: a review and practical guide. J. Cheminform. 12:56. doi: 10.1186/s13321-020-00460-5 33431035 PMC7495975

[B9] Esteva A. Robicquet A. Ramsundar B. Kuleshov V. DePristo M. Chou K. . (2019). A guide to deep learning in healthcare. Nat. Med. 25, 24–29. doi: 10.1038/s41591-018-0316-z 30617335

[B10] Feinberg E. N. Sur D. Wu Z. Husic B. E. Mai H. Li Y. . (2018). PotentialNet for molecular property prediction. ACS Cent. Sci. 4, 1520–1530. doi: 10.1021/acscentsci.8b00507 30555904 PMC6276035

[B11] Gawehn E. Hiss J. A. Schneider G. (2016). Deep learning in drug recommendation. Mol. Inform. 35, 3–14. doi: 10.1002/minf.201501008 27491648

[B12] Gilmer J. Schoenholz S. S. Riley P. F. Vinyals O. Dahl G. E. (2017). “Neural message passing for quantum chemistry,” in Proceedings of the International Conference on Machine Learning (Sydney), 1263–1272. Available online at: https://proceedings.mlr.press/v70/gilmer17a.html (Accessed Februrary 1, 2026).

[B13] Harbeck N. Penault-Llorca F. Cortes J. Gnant M. Houssami N. Poortmans P. . (2019). Breast cancer. Nat. Rev. Dis. Primers. 5:66. doi: 10.1038/s41572-019-0111-2 31548545

[B14] Hoghooghi Esfahani H. Toyonaga S. Oyibo K. (2025). The application of explainable artificial intelligence in the prediction, diagnoses, treatment, and management of chronic diseases: a systematic review. Digit Health. 11:20552076251355669. doi: 10.1177/20552076251355669 41312145 PMC12647564

[B15] Hu Q. Feng M. Lai L. Pei J. (2018). Prediction of drug-likeness using deep autoencoder neural networks. Front. Genet. 9:585. doi: 10.3389/fgene.2018.00585 30538725 PMC6277570

[B16] Hu Y. Maggiora G. Bajorath J. (2016). Comparing structural fingerprints using a literature-based similarity benchmark. J. Cheminform. 8:36. doi: 10.1186/s13321-016-0148-0 27382417 PMC4932683

[B17] Jumper J. Evans R. Pritzel A. Green T. Figurnov M. Ronneberger O. . (2021). Highly accurate protein structure prediction with AlphaFold. Nature. 596, 583–589. doi: 10.1038/s41586-021-03819-2 34265844 PMC8371605

[B18] Kearnes S. McCloskey K. Berndl M. Pande V. Riley P. (2016). Molecular graph convolutions: moving beyond fingerprints. J. Comput. Aided Mol. Des. 30, 595–608. doi: 10.1007/s10822-016-9938-8 27558503 PMC5028207

[B19] Kim S. Chen J. Cheng T. Gindulyte A. He J. He S. . (2019). PubChem 2019 update: improved access to chemical data. Nucl. Acids Res. 47, D1102–D1109. doi: 10.1093/nar/gky1033 30371825 PMC6324075

[B20] Lavecchia A. (2015). Machine-learning approaches in drug recommendation: methods and applications. Drug Discov. Today. 20, 318–331. doi: 10.1016/j.drudis.2014.10.012 25448759

[B21] Lavecchia A. Cerchia C. (2019). Recent advances in developing PCSK9 inhibitors for lipid-lowering Therapy. *Future Med. Chem*. 11, 423–441. doi: 10.4155/fmc-2018-0294 30892945

[B22] List of 96 Breast Cancer Medications Compared. (n.d.). Available online at: https://www.drugs.com/condition/breast-cancer.html (Accessed Februrary 1, 2026).

[B23] Liu M. Srivastava G. Ramanujam J. Brylinski M. (2024). SynerGNet: a graph neural network model to predict anticancer drug synergy. Biomolecules. 14:253. doi: 10.3390/biom14030253 38540674 PMC10967862

[B24] Mak K. K. Pichika M. R. (2019). Artificial intelligence in drug development: present status and future prospects. Drug Discov. Today. 24, 773–780. doi: 10.1016/j.drudis.2018.11.014 30472429

[B25] Mayr A. Klambauer G. Unterthiner T. Hochreiter S. (2016). DeepTox: toxicity prediction using deep learning. Front. Environ. Sci. 3:80. doi: 10.3389/fenvs.2015.00080

[B26] Nhlapho S. Nyathi M. Ngwenya B. L. Dube T. (2024). Druggability of pharmaceutical compounds using Lipinski rules with machine learning. Sci. Pharm. 3, 177–192. doi: 10.58920/sciphar0304264

[B27] Pan Y. Ren H. Lan L. Li Y. Huang T. (2023). Review of predicting synergistic drug combinations. Life. 13:1878. doi: 10.3390/life13091878 37763281 PMC10533134

[B28] PredictRx WebTool. Available online at: https://predictrx.streamlit.app/ (Accessed Februrary 1 2026).

[B29] Preuer K. Lewis R. P. I. Hochreiter S. Bender A. Bulusu K. C. Klambauer G. (2018). DeepSynergy: predicting anti-cancer drug synergy with deep learning. Bioinformatics. 34, 1538–1546. doi: 10.1093/bioinformatics/btx806 29253077 PMC5925774

[B30] Pun F. W. Ozerov I. V. Zhavoronkov A. (2023). AI-powered therapeutic target discovery. Trends Pharmacol. Sci. 44, 561–572. doi: 10.1016/j.tips.2023.06.010 37479540

[B31] Qureshi R. Irfan M. Gondal T. M. Khan S. Wu J. Hadi M. U. . (2023). AI in drug recommendation and its clinical relevance. Heliyon. 9:e17575. doi: 10.1016/j.heliyon.2023.e17575 37396052 PMC10302550

[B32] Rives A. Meier J. Sercu T. Goyal S. Lin Z. Liu J. . (2021). Biological structure and function emerge from scaling unsupervised learning to 250 million protein sequences. Proc. Natl. Acad. Sci. USA. 118:e2016239118. doi: 10.1073/pnas.2016239118 33876751 PMC8053943

[B33] Schneider P. Walters W. P. Plowright A. T. Sieroka N. Listgarten J. Goodnow R. A. Jr . (2020). Rethinking drug design in the artificial intelligence era. Nat. Rev. Drug. Discov. 19, 353–364. doi: 10.1038/s41573-019-0050-3 31801986

[B34] Schwaller P. Probst D. Vaucher A. C. Nair V. H. Kreutter D. Laino T. . (2021). Mapping the space of chemical reactions using attention-based neural networks. Nat. Mach. Intell. 3, 144–152. doi: 10.1038/s42256-020-00284-w

[B35] Segler M. Preuss M. Waller M. P. (2018). Planning chemical syntheses with deep neural networks and symbolic AI. Nature. 555, 604–610. doi: 10.1038/nature25978 29595767

[B36] Srivastava G. Malwe A. Sharma A. Shastri V. Hibare K. Sharma V. (2020). Molib: A machine learning based classification tool for the prediction of biofilm inhibitory molecules, Genomics, Vol. 112. 2823–2832doi: 10.1016/j.ygeno.2020.03.020 32229287

[B37] Stokes J. M. Yang K. Swanson K. Jin W. Cubillos-Ruiz A. Donghia N. M. . (2020). A deep learning approach to antibiotic discovery. Cell. 180, 688–702. doi: 10.1016/j.cell.2020.01.021 32084340 PMC8349178

[B38] Sung H. Ferlay J. Siegel R. L. Laversanne M. Soerjomataram I. Jemal A. . (2021). Global cancer statistics 2020. CA Canc. J. Clin. 71, 209–249. doi: 10.3322/caac.21660 33538338

[B39] Sutton R. T. Pincock D. Baumgart D. C. Sadowski D. C. Fedorak R. N. Kroeker K. I. (2020). An overview of clinical decision support systems: benefits, risks, and strategies for success. NPJ Digit. Med. 3:17. doi: 10.1038/s41746-020-0221-y 32047862 PMC7005290

[B40] Topol E. J. (2019). High-performance medicine: the convergence of human and artificial intelligence. Nat. Med. 25, 44–56. doi: 10.1038/s41591-018-0300-7 30617339

[B41] Vamathevan J. Clark D. Czodrowski P. Dunham I. Ferran E. Lee G. . (2019). Applications of machine learning in drug recommendation and development. Nat. Rev. Drug Discov. 18, 463–477. doi: 10.1038/s41573-019-0024-5 30976107 PMC6552674

[B42] Walters W. P. Murcko M. (2020). Assessing the impact of generative AI on medicinal chemistry. Nat. Biotechnol. 38, 143–145. doi: 10.1038/s41587-020-0418-2 32001834

[B43] Walters W. P. Wang R. (2020). New trends in virtual screening. J. Chem. Inf. Model. 60, 4109–4111. doi: 10.1021/acs.jcim.0c01009 32981325

[B44] Wang N. N. Zhu B. Li X. L. Liu S. Shi J. Y. Cao D. S. . (2023). Comprehensive review of drug–drug interaction prediction based on machine learning: current status, challenges, and opportunities. J. Chem. Inf. Model. 64, 96–109. doi: 10.1021/acs.jcim.3c01304 38132638

[B45] Wigh D. S. Goodman J. M. Lapkin A. A. (2022). A review of molecular representation in the age of machine learning. Wiley Interdiscip. Rev. Comput. Mol. Sci. 12:e1603. doi: 10.1002/wcms.1603

[B46] Winter R. Montanari F. Noé F. Clevert D. A. (2019). Learning continuous and data-driven molecular descriptors by translating equivalent chemical representations. Chem. Sci. 10, 1692–1701. doi: 10.1039/C8SC04175J 30842833 PMC6368215

[B47] Wishart D. S. Feunang Y. D. Guo A. C. Lo E. J. Marcu A. Grant J. R. . (2018). DrugBank 5.0: a major update to the DrugBank database for 2018. Nucl. Acids Res. 46, D1074–D1082. doi: 10.1093/nar/gkx1037 29126136 PMC5753335

[B48] Ying Z. Bourgeois D. You J. Zitnik M. Leskovec J. (2019). GNNExplainer: generating explanations for graph neural networks. Adv. Neural Inf. Process Syst. 32. 32265580 PMC7138248

[B49] Zagidullin B. Aldahdooh J. Zheng S. Wang W. Wang Y. Saad J. . (2019). DrugComb: an integrative cancer drug combination data portal. Nucl. Acids Res. 47, W43–W51. doi: 10.1093/nar/gkz337 31066443 PMC6602441

[B50] Zhang G. Xue Z. Yan C. Wang J. Luo H. (2021). A novel biomarker identification approach for gastric cancer using gene expression and DNA methylation dataset. Front. Genet. 12:644378. doi: 10.3389/fgene.2021.644378 33868380 PMC8044773

[B51] Zhao H. Zhong J. Liang X. Xie C. Wang S. (2025). Application of machine learning in drug side effect prediction: databases, methods, and challenges. Front. Comput. Sci. 19:195902. doi: 10.1007/s11704-024-31063-0

[B52] Zhou Z. H. (2021). Machine Learning. Singapore: Springer Nature. doi: 10.1007/978-981-15-1967-3

[B53] Zitnik M. Agrawal M. Leskovec J. (2018). Modeling polypharmacy side effects with graph convolutional networks. Bioinformatics. 34, i457–i466. doi: 10.1101/258814 29949996 PMC6022705

